# Flexible Thin-Film PZT Ultrasonic Transducers on Polyimide Substrates

**DOI:** 10.3390/s21031014

**Published:** 2021-02-02

**Authors:** Tianning Liu, Ajay Dangi, Jeong Nyeon Kim, Sri-Rajasekhar Kothapalli, Kyusun Choi, Susan Trolier-McKinstry, Thomas Jackson

**Affiliations:** 1 School of Electrical Engineering and Computer Science, Penn State University, University Park, PA 16802, USA; kxc104@psu.edu (K.C.); set1@psu.edu (S.T.-M.); tnj1@psu.edu (T.J.); 2 Department of Biomedical Engineering, Penn State University, University Park, PA 16802, USA; axd571@psu.edu (A.D.); szk416@psu.edu (S.-R.K.); 3 Department of Engineering Science and Mechanics, Penn State University, University Park, PA 16802, USA; juk252@psu.edu; 4 Department of Materials Science and Engineering, Penn State University, University Park, PA 16802, USA

**Keywords:** flexible transducer, PZT pMUT, thin-film transducer, flexible PZT, flexible pMUT, solution-cast polyimide, anisotropic conductive film

## Abstract

We report flexible thin-film lead zirconate titanate (PZT)-based ultrasonic transducers on polyimide substrates. The transducers are bar resonators designed to operate in the width extension mode. The active elements are 1 µm thick PZT films that were crystallized on Si substrates at 700 °C and transferred to 5 µm thick solution-cast polyimide via dissolution of an underlying release layer. Underwater pitch–catch testing between two neighboring 100 µm × 1000 µm elements showed a 0.2 mV signal at a 1.5 cm distance for a driving voltage of 5 V peak at 9.5 MHz. With the same excitation, a 33 kPa sound pressure output at a 6 mm distance and a 32% bandwidth at −6 dB were measured by hydrophone.

## 1. Introduction

There is growing demand for flexible, miniaturized, ultrasonic transducer devices in medical diagnostics and biometric authentication applications. Compared with conventional ultrasound technologies, such devices can conform to complex shapes and geometries, integrate with portable electronics operating at a low driving voltage, and access small apertures of imaging interest. For example, miniaturized ultrasonic transducer devices would be particularly helpful in catheter-based ultrasound imaging technology, and would facilitate development of either forward-looking or side-looking imagers in which the transducer is wrapped around a central support ~1 mm in diameter. The operating frequency requirement for intravascular ultrasound (IVUS) that would benefit from such a flexible transducer is approximately between 10 MHz and 40 MHz [1,2,3,4,5]. Wearable ultrasonic systems attached to the human body would enable more personalized and adaptive medical diagnoses and treatments, such as for neuromodulation [6].

Another application of interest that would benefit from flexible transducers is finger vein detection. Authentication by imaging finger veins has advantages relative to other biometrics as the results are more accurate, difficult to forge, and less affected by epidermal and environmental conditions [7]. Current commercial finger vein sensors have relatively large footprints, as they are limited to using infrared light combined with a charge-coupled device (CCD) camera [8]. Development of piezoelectric thin-film-based ultrasonic transducers will enable this authentication technology to be integrated into more portable electronics, as well as allow a broader scope of applications.

Historically, flexible transducers have been predominantly based on polymeric piezoelectrics, such as polyvinylidene fluoride (PVDF) and composite piezoelectric materials [9,10,11,12,13,14]. Despite their flexibility, the effectiveness of these materials is limited by a low piezoelectric response and a low maximum operating temperature.

As an alternative, inorganic piezoelectric thin films can be integrated with metallic foils or polymeric substrates for improved electromechanical responses while realizing mechanical flexibility. Because many of the inorganic piezoelectric materials require a crystallization temperature (e.g., PZT, Pb(Mg_1/3_Nb_2/3_)O_3_-PbTiO_3_, and BaTiO_3_) substantially higher than the thermal limit of nearly all polymeric materials, thermally robust metallic foils provide some advantages for flexible device fabrication. Nickel and stainless-steel foils have been reported as flexible substrates for PZT-based MEMS devices due to their high mechanical and thermal robustness [15,16,17,18,19]. Despite being thermally robust and mechanically ductile, metal foils are stiffer than polymers and often require a buffer layer for insulation and/or passivation. Using polymer as the flexible substrate can provide important advantages for MEMS applications. There are, however, very few literature examples reporting flexible piezoelectric thin-film ultrasonic transducers on polymeric substrates. Information on design limitations, processing challenges, and the acoustic performance for such devices is not readily available to the ultrasound community. In addition, the paper reports the use of anisotropic conductive film bonding as a robust approach to making electrical connections to the flexible transducers.

In this study, flexible transducers were designed and fabricated using 1 µm thick lead zirconate titanate (PZT) thin films crystallized at 700 °C with an underlying 150 nm thick ZnO release layer. The devices were fabricated on Si and transferred to 5 µm thick solution-cast polyimide substrates by etching away the ZnO layer in acetic acid. The transducer elements were 100 µm × 1000 µm individual bar resonators accessed by top and bottom Pt and Au electrodes, and they were designed to operate in the width extension mode. Electrical connections were made by bonding flexible ribbon cables with anisotropic conductive films (ACFs).

## 2. Device Fabrication

**Figure 1:**
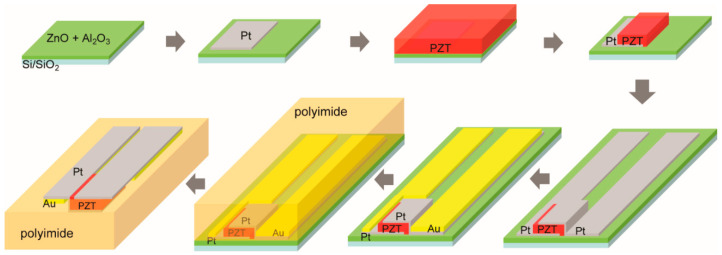
Process for fabricating flexible thin-film PZT bar resonator-type transducers on a few micron thick polyimide substrate.

The fabrication procedure for flexible transducers is based on a previously reported release-and-transfer process [20], with a few modified and additional steps. The process flow is illustrated schematically in Figure 1. To fabricate the flexible ultrasound transducers, a 150 nm thick ZnO release layer was deposited by plasma-enhanced atomic layer deposition (PEALD) at 200 °C on a 100 mm silicon wafer with 1 µm thick thermal SiO_2_ using diethylzinc (DEZ) as the precursor and N_2_O as the oxidant. The ZnO release layer was capped with a 10 nm thick PEALD Al_2_O_3_ diffusion barrier layer grown at 200 °C using trimethylaluminum (TMA) as the precursor and CO_2_ as the oxidant. The oxide film thickness was confirmed by spectroscopic ellipsometry measurement (RC2, J.A. Woollam, Lincoln, NE, USA).

After deposition of the release layer and diffusion barrier, a 150 nm thick platinum layer was deposited by DC magnetron sputtering and patterned by lift-off to define the first electrode layer for the PZT transducers. The platinum was deposited at room temperature in a Kurt J. Lesker CMS 18 system (Pittsburgh, PA, USA) at a 2.5 mTorr process pressure with an RF power at 200 W. The 3 inch Pt target was tilted at 15° and placed at a 15 cm distance from the substrate in a sputter-up configuration.

Next, a 1 µm thick PbZr_0.52_Ti_0.48_O_3_ (PZT) layer with 2% Nb doping was deposited over the patterned Pt contact using chemical solution deposition by spin-coating. The PZT was crystallized in air at 700 °C in an AccuThermo AW810 rapid thermal annealing (RTA) system (Allwin21 Corp, Morgan Hill, CA, USA). The as-crystallized film was approximately randomly oriented. The PZT layer was then patterned into individual bar resonators using a high-density plasma etching system NE-550 (ULVAC, Inc., Methuen, MA, USA). A 10 µm thick positive photoresist AZ 4620 (MicroChemicals GmbH, Ulm, Germany) was used as the soft etch mask. The etch process took place at 3.5 mTorr and 200 V substrate self-bias with a gas mixture of Ar, CF_4_, and Cl_2_ in the ratio of 15:28:7. To prevent overheating and potentially crosslinking the photoresist, the etching process was carried out in multiple cycles of an etch-pause sequence (a 60 s pause between 20 s etching steps) with constant helium backside cooling. These parameters gave an etch rate of ~80 nm/min for the PZT and ~800 nm/min for the photoresist. The thickness of the PZT and the photoresist layer was monitored by ellipsometry (8000× SE, Nanometrics Inc., Milpitas, CA, USA) and contact profilometry (P16+, KLA Corporation, Milpitas, CA, USA), respectively. After etching, the photoresist was removed in N-Methyl-2-Pyrrolidone (NMP) at ~70 °C for 20 min followed by an isopropanol (IPA) and a deionized water rinse before the sample was blown dry. To remove any organic residue, an oxygen plasma ash was performed in a 150 mm electrode PT720 parallel-plate plasma etcher (PlasmaTherm, Fremont, CA, USA) at 100 mTorr and 100 V self-bias for 5 min. Post-etch annealing was carried out at 550 °C in air in an RTA system to remove etch-induced damage.

**Figure 2:**
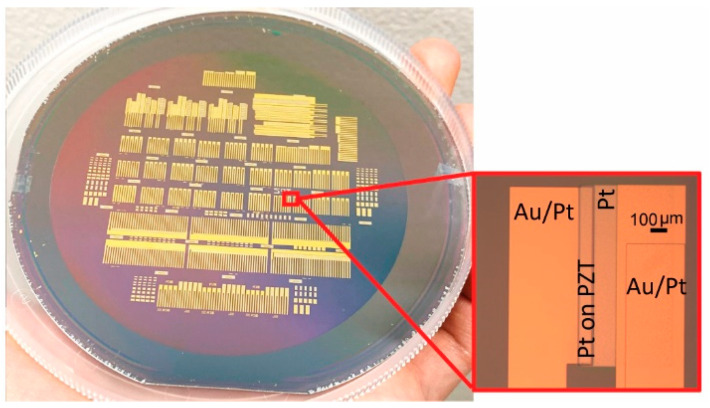
PZT transducer elements, arranged in 1-D arrays, as well as independently accessed by top and bottom Pt and Au electrodes, are fabricated on a 100 mm Si wafer prior to release. A close-up of an individual element is shown in the inset.

After the PZT dry-etch, a second 150 nm Pt electrode layer was deposited by sputtering and patterned by lift-off following the same procedure as the first layer. The second Pt deposition was also used to form a 3.5 mm long and 250 µm wide contact in preparation for ACF bonding flexible cables after release. Five-hundred nanometer thick Au was then deposited by DC magnetron sputtering in a Kurt J. Lesker CMS 18 system, and patterned by lift-off to reduce series resistance and allow for high-frequency operation. Contact profilometry was used to confirm the film’s thickness for both Pt and Au. The PZT area was excluded from Au deposition to avoid any undesirable bending modes as a result of structural asymmetry. The wafer prior to the polyimide processing is shown in Figure 2. The wafer is populated with individual transducers arranged in a 1-D array and individual elements independently accessed with top and bottom leads. The microscope image zooms in on a single transducer element that is accessed by top and bottom Pt contacts, as well as the overlapping Au layer.

Polyimide was chosen as the flexible substrate for its high thermal and chemical robustness and low thermal expansion coefficient. The fabricated structure could therefore withstand high-temperature poling and operation (PI-2611 service temperature ~360 °C), remain undamaged in the acetic acid etchant during release and post-processing solvent cleaning, and promote good adhesion to other thin-film layers in the device stack. To complete the flexible transducers, a 50 nm thick Al_2_O_3_ layer was deposited by PEALD at 200 °C using TMA as the precursor and CO_2_ as the oxidant. To form the polyimide transfer substrate, PI-2611 poly(amic acid) solution (HD MicroSystems) was spun onto the Al_2_O_3_-protected sample at 2000 RPM in air, baked on a hotplate at 110 °C for 5 min in air, and transferred to a tube furnace for curing in an inert environment—the chamber was purged with Ar at 4 SLM for 5 min after loading the sample. To cure the poly(amic acid) layer, the furnace temperature was ramped to 305 °C at a 4 °C/min rate, then held at 305 °C for 75 min. The film thickness of the cured polyimide was measured to be 5 µm by contact profilometry. The inert curing ambient and the Al_2_O_3_ layer prevent PZT degradation by reaction products of the PI-2611 imidization. PZT films cured in air without the Al_2_O_3_ barrier layer suffered a large reduction (80–90%) of switchable polarization and a relatively smaller reduction of permittivity (40–50%), similar to the degradation observed for PZT/Pt capacitors annealed in H_2_ [21,22,23,24]. We speculate that water generated by the poly(amic acid) imidization reacts with the Pt electrodes to generate hydrogen, thus degrading the PZT polarization. For structures with a blanket PZT and a blanket bottom Pt electrode, as demonstrated in the previous report [20], the degradation could be effectively prevented by only adding an Al_2_O_3_ barrier layer over the Pt while the PI-2611 precursor was cured in air. Here, however, the patterned Pt and PZT combination in the transducer structure would allow the hydrogen to access the PZT, and the inert curing environment was needed to prevent degradation. In addition to acting as a barrier layer, the blanket Al_2_O_3_ also served to stiffen the released structure, reducing handling difficulty and preventing potential damage to the elements from excessive flexing. A third function of the Al_2_O_3_ was to promote adhesion between the polyimide and the metal contacts.

Finally, the PZT–polymer combination was released by wet etching the ZnO release layer at 50 °C in 50% acetic acid. Mechanical agitation such as stir-bar use was avoided to prevent damage to the flexible structure. Diluting (reducing the viscosity) and heating the etchant assisted the mass transport by encouraging the exchange of reaction products and fresh etchant at the exposed ZnO edges. Acetic acid at a higher concentration does not necessarily yield a faster release, due to the slow mass transport resulting from the high viscosity. The released sample was dried in ambient air.

Electrical connections were made using flexible cables bonded to the transducers with an anisotropic conductive film (ACF). ACF bonding is widely used in chip-on-film (COF) and chip-on-glass (COG) applications, such as in flat panel display assembly [25,26,27]. The cables used in this study were a 50 µm thick polyimide base with 3 µm thick copper traces that were gold-plated to avoid oxidation. The metal traces had the same pitch dimension (0.5 mm) as the contact leads on the flexible sample. The ACF, a transparent B-stage epoxy matrix embedded with conductive particles, served as a conducting adhesive between the cable and the transducer sample. An 18 µm thick and 1.5 mm wide AC-7206U ACF (Hitachi, Tokyo, Japan) was first tacked on the cable with ~1 MPa of pressure at 90 °C for 10 s using a UNITEK 9200 bonder paired with a Hughes HTT-1000 power supply. Next, the cable with the ACF was placed on the flexible sample with traces aligned to the transducer contacts. Next, ~1 MPa of pressure was applied to the sample/ACF/cable sandwich structure at 180 °C for 25 s to deform and cure the epoxy, trapping the conductive particles between each aligned electrode pair to establish electrical connections while maintaining electrical insulation in regions outside the electrodes. Mechanical bonding was achieved once the epoxy of ACF was cured. Bonding flexible cables with ACF is an effective alternative to the wire bonding technique, especially for flexible devices.

**Figure 3:**
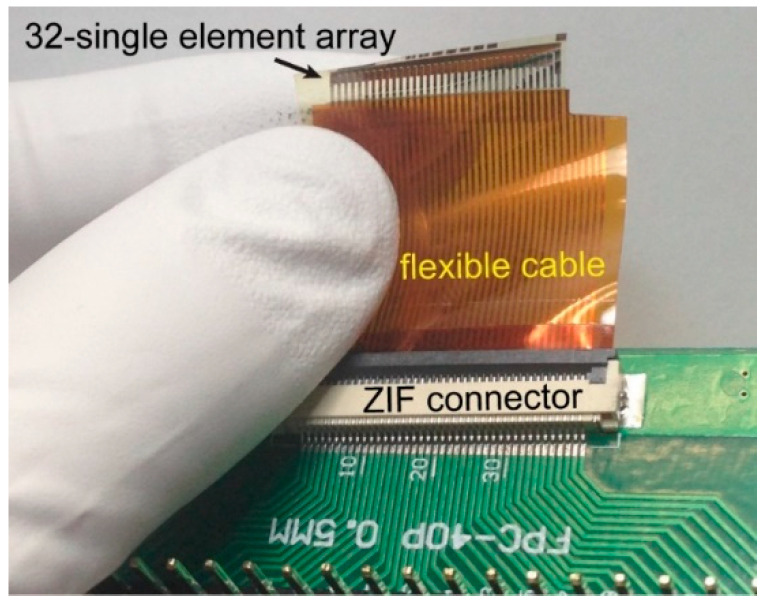
1-D array of 32 single element PZT thin-film transducers on a 5 µm polyimide layer. A flexible cable connects the transducer elements to a printed circuit board. Anisotropic conductive film (ACF) bonding was used to join the cable and the flexible sample while the cable was inserted to a zero insertion force (ZIF) connector.

The completed transducers were coated with a 1.6 µm thick parylene C layer for waterproofing. The polymer coating was deposited using a LabTop parylene deposition system (Para Tech Coating, Aliso Viejo, CA, USA). The flexible cables were inserted into zero insertion force (ZIF) connectors mounted on printed circuit boards. The connections on the circuit board were then fanned-out to soldered pin-headers. This connection setup can be seen in Figure 3, which shows a 1-D transducer array of 32 single elements sharing a common ground. Each element has a 1 µm thick PZT layer of 100 µm × 1000 µm in the lateral dimension with 400 µm of spacing between adjacent elements. The transducer elements were poled at three times the coercive field, or 130 kV/cm, for 15 min at 120 °C.

## 3. Acoustic Testing

### 3.1. Pitch–Catch

Pitch–catch testing was performed in water with two neighboring single elements in transmit and receive, respectively. The acoustic waves transmitted by the pitch element were reflected from an aluminum plate placed at a 1.5 cm distance and received by the catch element. The fabricated flexible transducer sample was mounted to a kinematic mirror mount on a post fixed on an NRT 100 three-axis linear translation stage (Thorlabs, Inc., Newton, NJ, USA). The kinematic mount allowed for adjustment of the orientation of the transducer about the axes of rotation in the pitch (vertical) and yaw (horizontal) directions. The transducers were attached on the movable front plate of the mount, which was driven by rotating the screws on the fixed back plate.

**Figure 4:**
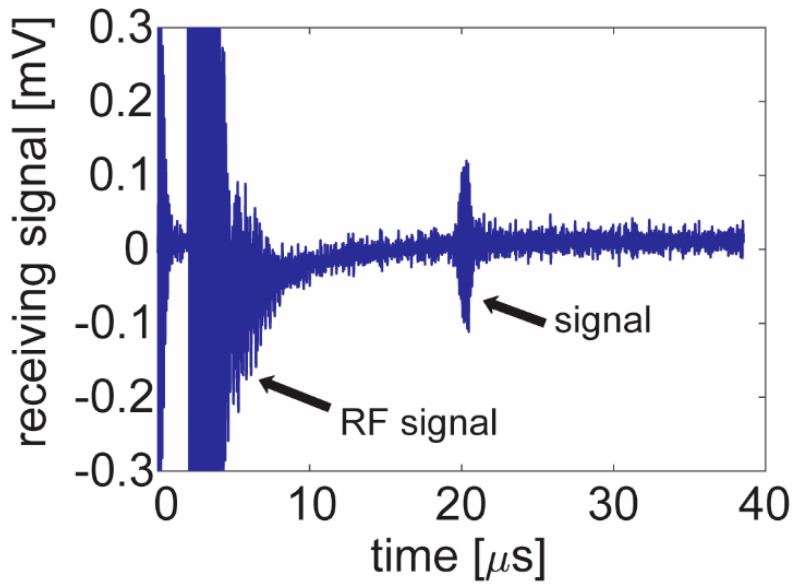
Pitch–catch testing of two neighboring 100 µm × 1000 µm single elements transmitting and receiving an acoustic signal reflected from a metal reflector at a 1.5 cm distance. The transmitting element was driven by a 5 V_p_ 5-cycle unipolar excitation and the receiving element detected a 0.2 mV signal.

The transmitting element was driven by a SIGLENT SDG 6022X (Solon, OH, USA) function generator in burst mode with a unipolar five-cycle 5 V peak excitation. The reflected sound wave from the aluminum plate was detected by the receiving element, and the signal was amplified by an Olympus 5072 PR ultrasound pulser/receiver and measured by a Tektronix MDO 3024 oscilloscope (Beaverton, OR, USA). Typically, 30–39 dB amplification was used for the flexible PZT resonators. The data presented in this study are the back-calculated voltage levels after removing the gain. The maximum signal detected by the receiving element was 0.2 mV (Figure 4) observed at 9.5 MHz. The signal level in Figure 4 is a result of a single-element pitching and a single-element catching.

This observed frequency was compared with a theoretical resonance frequency value derived for a free-standing PZT ceramic plate. Operating in the fundamental width extension mode with free boundary conditions, the resonance frequency *f_r_* is a function of the sound velocity *v* in PZT and the width dimension *W*, expressed in (1) [28]

(1) $$f_{r}=\frac{v}{2W}.$$

Using 4300 m/s as the approximate sound velocity [29], the estimated resonance frequency for a 100 µm wide flexible thin-film PZT resonator is 21.5 MHz, more than two times larger than the experimental maximum signal frequency. The discrepancy is presumably a result of additional mass-loading associated with the electrodes and polyimide, introduction of a flexural mode, and/or the frequency response of the test system. In terms of the mass-loading case, considering the model of a simple harmonic vibration to a first approximation [30], as shown in (2), the resonance frequency $f_{r}$ can be written as

(2) $$f_{r}=\frac{1}{2\pi }\sqrt{\frac{k}{m}},$$

where *k* is the spring constant, and *m* is the mass. For the fabricated transducer element, the combined mass of the polyimide and the dense Pt contributed to approximately half of the total mass; this would lower the resonance frequency by a factor of ~1.4.

### 3.2. Hydrophone Measurement

**Figure 5:**
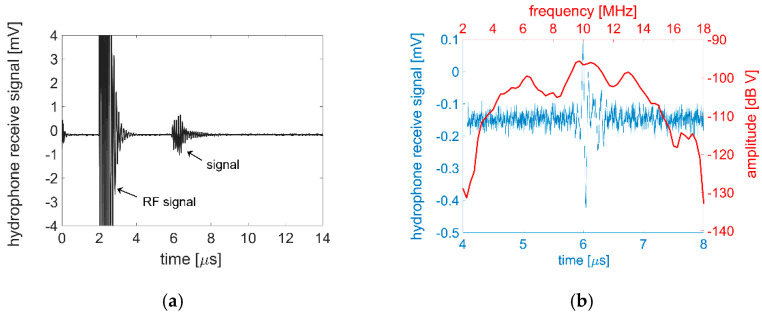
**(a)** Hydrophone characterization of a 100 µm × 1000 µm single element driven with a 5 V unipolar sinusoidal excitation at 9.5 MHz. The detected 1.6 mV corresponds to a 33 kPa sound pressure output; (**b**) Hydrophone detection of a single cycle excitation along with FFT calculation, which shows a −6 dB bandwidth of ~32%.

The sound pressure output and bandwidth were evaluated with a capsule-type hydrophone (HGL-0085, Onda Inc., Sunnyvale, CA, USA) placed at a 6 mm distance from the transducer elements. The hydrophone was fixed on a kinematic mirror mount used for the flexible transducer, and the detected signal was measured by an oscilloscope (Tektronix MDO 3024). With a single transducer element driven with five cycles of 5 V unipolar excitation, the hydrophone was able to detect a 1.6 mV signal with only the hydrophone’s internal amplification, as shown in Figure 5a. The voltage level corresponds to a 33 kPa sound pressure level.

Due to the novelty of the polymer-substrate-based piezoelectric transducer, studies specifically on pressure output are not readily available in the literature. Comparison was therefore made with recent rigid substrate-based piezoelectric micromachined ultrasonic transducers (PMUTs), as shown in Table 1. The column for the normalized pressure output indicates that the transducers in this study achieved relatively high performance under a low driving voltage compared with similar devices reported recently.

Using the same electrical excitation, but with a one-cycle burst at 9.5 MHz, bandwidth was evaluated as 32% at −6 dB through converting the detected signal from time to frequency domain using a fast Fourier transform (FFT), as shown in Figure 5b.

The reproducibility of the acoustic measurement results largely depended on the integrity of the alignment between the flexible transducers and the testing unit (i.e., metal plate and hydrophone). It is therefore important to ensure a consistent mechanism to finely align the transmitting and receiving units in both linear and rotational orientation.

## 4. Conclusions

Flexible bar resonator-type transducers were fabricated using 1 µm thick high-temperature-crystallized PZT films on a few micron thick polyimide substrates. To prevent degradation in the PZT films caused by the imidization process of the poly(amic acid), the precursor was cured in an inert Ar ambient over a 50 nm Al_2_O_3_ barrier layer. Flexible ribbon cables were fabricated and bonded to the released transducers and inserted into a ZIF connector on a printed circuit board to establish electrical connections. Underwater pitch–catch testing between two neighboring 100 µm × 1000 µm elements with a metal reflector at a 1.5 cm distance showed a 0.2 mV signal for a unipolar excitation of 5 V peak at 9.5 MHz. Driven by the same excitation, a 33 kPa sound pressure output at a 6 mm distance and a 32% bandwidth at −6 dB was measured by a hydrophone. To resolve the discrepancy between the theoretical and experimental central frequency results, ongoing work is directed towards developing a more accurate model using finite element analysis (FEA) that incorporates information about the entire device stack and the operating environment to replace the simple free-standing plate model. In addition, laser vibrometry measurements are planned to clarify the vibration mode of the flexible transducers and serve as a crosscheck of the FEA model. The transducers reported in this study are the first high-temperature-crystallized thin-film PZT ultrasonic transducers transferred over large areas to flexible polymeric substrates. The development of flexible PZT devices can enable multiple applications that are inaccessible by conventional bulk or rigid substrate thin-film devices.

## Figures and Tables

**Table 1:** Pressure output compared with rigid substrate-based piezoelectric micromachined ultrasonic transducer (PMUT) devices.

Structure	Frequency	Pressure Output	Pressure Output/1000 µm^2^	Distance	Driving Voltage	Source
Flexible PZT bar resonator	9.5 MHz	33 kPa	0.33 kPa	6 mm	5 V	This work
Epitaxial PZT pMUT	8.5 MHz	6.3 kPa	2.65 kPa	max output	5 V	[31]
PZT pMUT	8 MHz	9.5 kPa	0.16 kPa	7.5 mm	5 V	[32]
AlN pMUT	20 MHz	25 kPa	0.12 kPa	0.8 mm	24 V	[33]

## Data Availability

The corresponding author will provide data on reasonable request due to restrictions. The data are not publicly available due to funding agent policies.
